# A novel *SIGMAR1* missense mutation leads to distal hereditary motor neuropathy phenotype mimicking juvenile ALS: a case report of China

**DOI:** 10.3389/fgene.2025.1477518

**Published:** 2025-04-16

**Authors:** Qinglong Yu, Risna Begam Mohammed Nazar, Sihui Chen, Qiaoling Qian, Junhui Wang, Xueping Chen

**Affiliations:** ^1^ Department of Neurology,Affiliated Hospital of Panzhihua University, Panzihua, China; ^2^ Department of Neurology, West China Hospital, Sichuan University, Chengdu, Sichuan, China; ^3^ Department of Neurology, Mount Sinai Hospital, Toronto, ON, Canada; ^4^ Sunsimiao Hospital, Beijing University of Chinese Medicine, Tongchuan, Shanxi, China

**Keywords:** SIGMAR, JALS, dHMN, diagnosis, case report

## Abstract

We present the case of a 16-year-old East Asian Chinese girl with a novel mutation in the *SIGMAR1* gene, initially diagnosed as juvenile amyotrophic lateral sclerosis (JALS). At the age of five, she began to exhibit gait abnormalities while walking, a condition that persisted for 4 years until muscle weakness and atrophy emerged, predominantly affecting her distal muscles symmetrically. Electromyography (EMG) initially revealed early abonormal motor conduction, and subsequent examinations indicated neurogenic damage accompanied by localized denervation potentials. Whole-exome sequencing identified compound heterozygous mutations in the *SIGMAR1* gene. Throughout the course of her illness, the patient exhibited slow disease progression without cognitive impairment or scoliosis development. We ultimately revised the diagnosis to distal hereditary motor neuropathy (dHMN). This study reports the case of *SIGMAR1* new locus mutation leading to dHMN in China, contributing to the expansion of the dHMN genetic database. In our patient, the initial EMG findings indicated issues with neurogenic conduction, followed by a slow progression of the disease. Subsequently, EMG results revealed axonal damage and denervation potentials. These clinical features can easily lead to confusion with JALS. This insight is valuable for improving diagnostic accuracy and understanding the clinical spectrum of dHMN related to *SIGMAR1* mutations.

## Introduction

Distal hereditary motor neuropathy (dHMN) is a relatively new and debated condition characterized by clinical and genetic heterogeneity (Li et al., 2015). It commonly presents with progressive distal muscle weakness and atrophy, sometimes accompanied by pyramidal signs, but notably lacks sensory abnormalities (Li et al., 2015; Ma et al., 2020; Tazir and Nouioua, 2024). To date, approximately 30 genes have been identified in association with dHMN, yet together they explain fewer than one-third of the observed cases (Ververis et al., 2020). Notably, the *SIGMAR1* gene is especially contentious due to significant phenotypic overlap; mutations within *SIGMAR1* are frequently associated with conditions that exhibit clinical features reminiscent of Amyotrophic Lateral Sclerosis (ALS) (Al-Saif et al., 2011; Luty et al., 2010; Li et al., 2023; Izumi et al., 2018; Kim et al., 2014). ALS is a fatal neurodegenerative disorder characterized by the progressive degeneration of upper and lower motor neurons (Feldman et al., 2022). When symptom onset occurs before the age of 25, the condition is classified as Juvenile ALS (JALS) (Lehky and Grunseich, 2021). However, “JALS” cases diagnosed due to *SIGMAR1* gene mutations do not typically exhibit the rapid progression and widespread neurogenic damage characteristic of ALS, leading to considerable confusion among clinicians and the families of patients.

We reported on an adolescent patient with involvement of both upper and lower motor neurons who harbors a novel locus variant in the *SIGMAR1* gene.

## Case presentation

At the age of 5, the patient began experiencing difficulties with walking, characterized by increased muscle tone in the legs and a tendency to fall while running quickly. Despite these symptoms being relatively mild and not significantly interfering with her daily activities, no medical evaluation was sought at that time. By the age of 9, she developed slight weakness in the distal upper limbs, reduced range of motion in her arms, and upper limb tremors when gripping objects. These symptoms progressively worsened over time, resulting in more frequent falls and diminished performance in sports activities. She did not report any symptoms related to fasciculations, choking, swallowing difficulties, respiratory challenges, and cognitive impairment. The patient went to the local hospital for medical help. Neurological examination revealed muscle atrophy in the limbs, particularly in the interosseous muscles of the hands. A postural tremor was observed in the left upper limb and muscle strength was nearly normal, with only a positive result for the Clipper Paper Test and a slight limitation in standing on tiptoes noted. Hyperactive tendon reflexes were present in the limbs, and the Babinski sign was positive. The EMG examination revealed decreased amplitudes of compound muscle action potential (CMAP) waves and motor nerve conduction abnormalities, including extended distal latencies and reduced F-wave frequencies. However, the initial EMG did not demonstrate spontaneous potentials or evidence of chronic denervation in the upper limbs (Supplementary Table S1). The patient was diagnosed with clinically “possible ALS” at that time in local hospital, although her EMG was not consistent with classic ALS manifestations.

**FIGURE 1:**
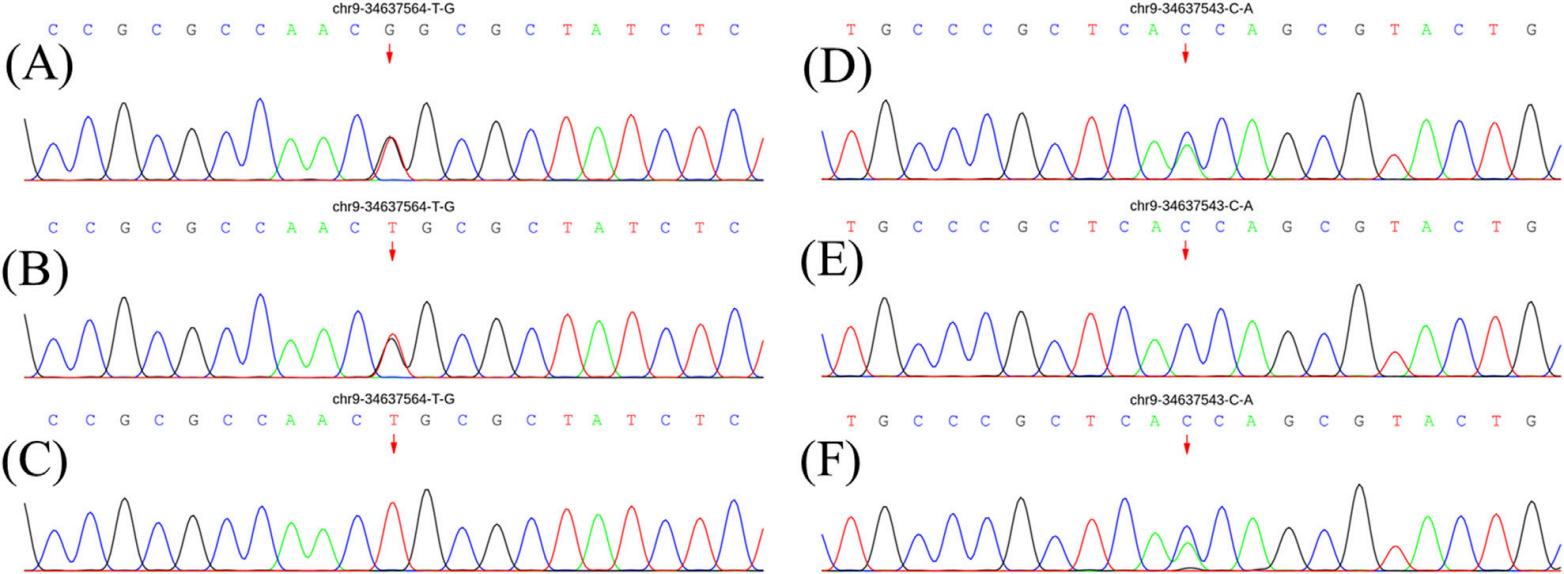
Genetic results for patients and parents. **(A)** and **(D)** show electrophoresis images of the proband. **(B)** and **(E)** show electrophoresis images of her father. **(C)** and **(F)** show electrophoresis images of her mother. The pathogenic gene mutation is consistent with autosomal recessive inheritance (compound heterozygous mutation pattern).

**FIGURE 2:**
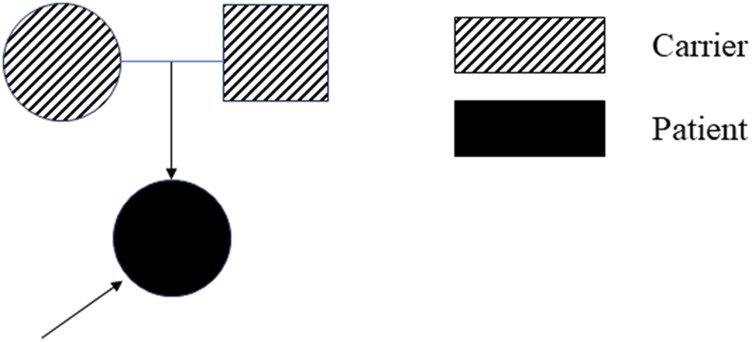
Diagram of family tree.

At age 14, the subsequent EMG revealed neurogenic damage affecting both the upper and lower limbs was observed, with evident axonal involvement, and a notable finding was the presence of abundant fasciculation potentials in the bilateral anterior tibialis muscles. Both ulnar and sural nerve conduction velocities were found to be decreased upon re-examination (Supplementary Table S2). At this time, her symptoms remained stable except for a slight reduction in lower limb muscle strength to grade 4+. Whole exome sequencing identified a compound heterozygous mutation in the *SIGMAR1* gene (Figure 1). The first gene mutation site [NM_005866. 2: 131A > c (p.Gln 44 Pro)] came from her father, and this mutation was not listed in all gene database, and the mutation frequency among the general populace being 0%. Analytical predictions employing multiple statistical methodologies indicated a detrimental impact on the gene and its protein products. Consequently, this mutation satisfied the classification for “possible pathogenic gene variation” (PM2+PM3+PP3+PP4). The other *SIGMAR1* gene variant [NM_005866.2: 151 + 1G>T] was inherited from her mother which has been shown to impair gene function *in vitro* and *in vivo*, and therefore met the standard of “pathogenic gene variant” (Richards et al., 2015). Notably, the patient’s parents exhibited no phenotypical manifestations of the disease (Figure 2). At this point, the patient was diagnosed with “clinically probable ALS”, considering the clinical presentation of both upper and lower motor neuron involvement coupled with genetic corroboration, although her EMG was not consistent with classic ALS manifestations (Shefner et al., 2020). The treatment regimen involved administering riluzole at a dose of 50 mg twice daily, accompanied by routine follow-up appointments.

**FIGURE 3:**
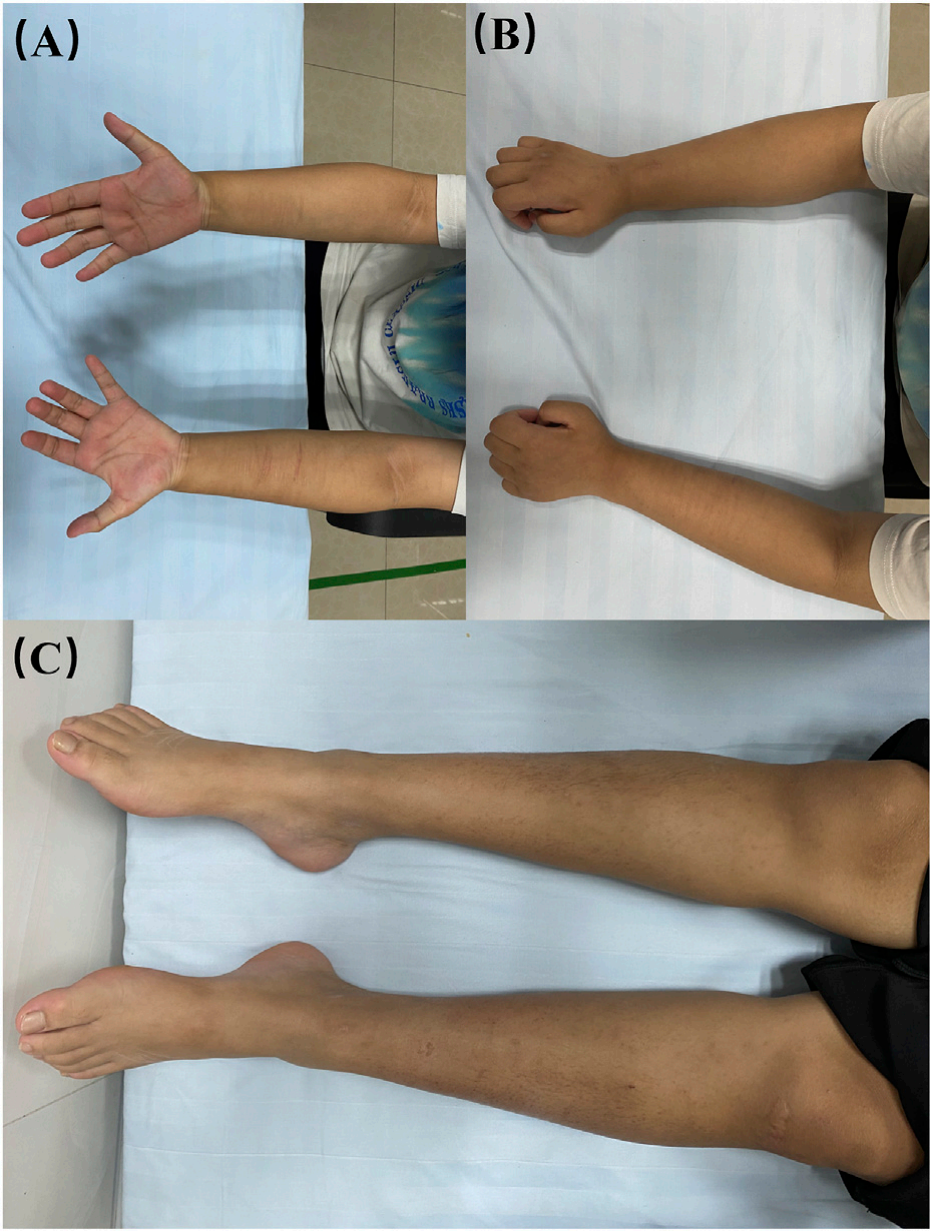
Visualisation of the patient’s limbs. **(A, B)** bilateral forearms; **(C)** bilateral lower limbs.

**FIGURE 4:**
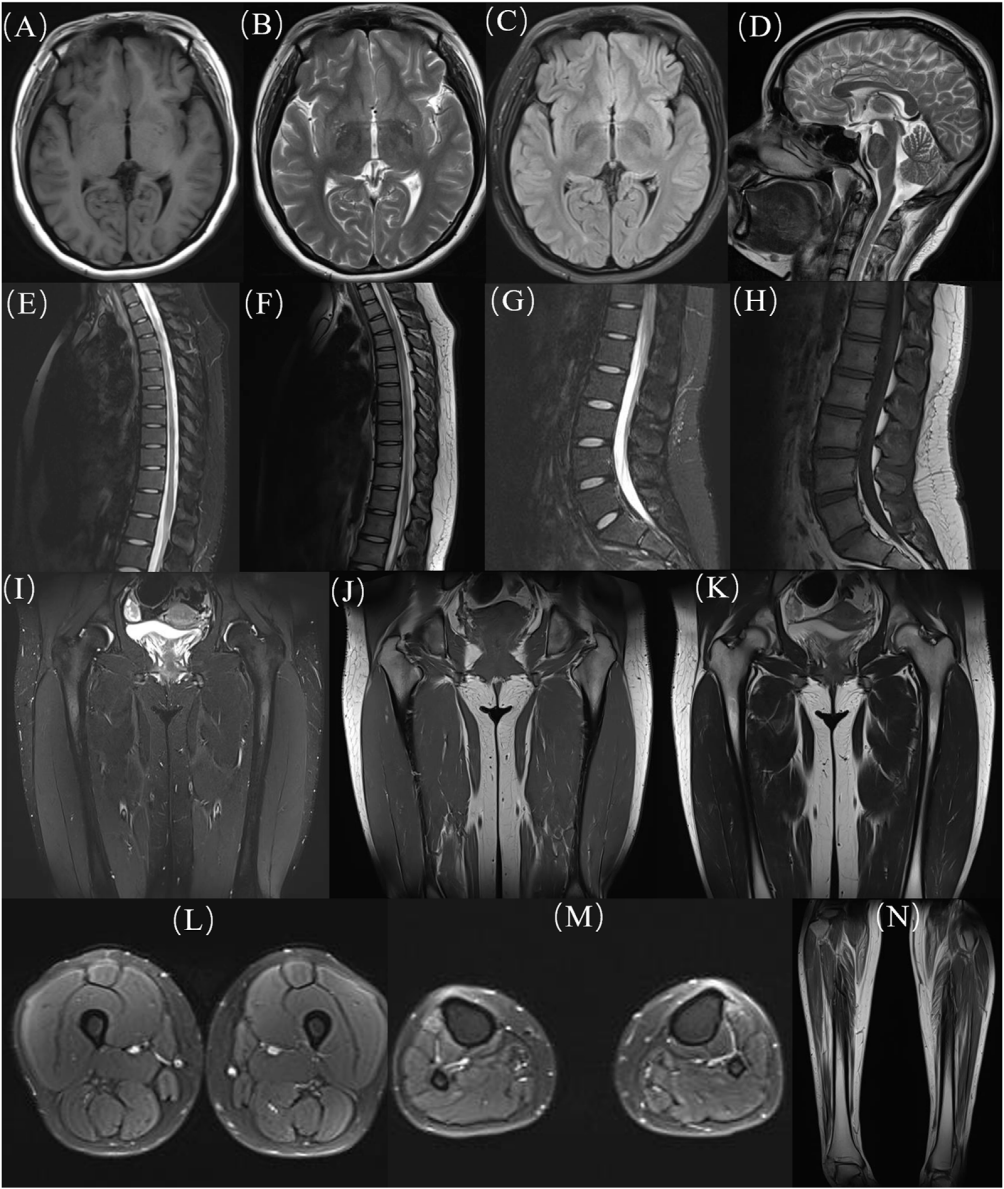
MRI images of the patient. **(A-D)** head, **(E, F)** thoracic segments; **(G, H)** lumbar segments; **(H-K)** hips; **(L)** thighs; **(M, N)** calves. MRI: Magnetic resonance imaging.

At age 16, during a follow-up visit, we found that her clinical symptoms remained stable, and no further progression of muscle atrophy was observed. Symmetrical muscle atrophy was observed in the distal extremities, with the lower limbs being more severely affected (Figure 3). We conducted a cognitive assessment, which revealed a Montreal Cognitive Assessment (MoCA) score of 23, a Mini-Mental State Examination (MMSE) score of 26, and a Frontal Assessment Battery (FAB) score of 14, indicating normal cognitive function. Furthermore, her brain magnetic resonance imaging was unremarkable, and no scoliosis was observed (Figure 4). The patient’s MRI of both lower legs exhibited noticeable bilateral calf muscle atrophy, which was symmetric, with areas showing infiltration of white adipose tissue (Figures 4M, N). Somatosensory evoked potential (SEP) and motor evoked potential (MEP) showed no significant abnormalities. Unfortunately, the patient declined the muscle biopsy due to concerns about invasive procedures. Based on the clinical features of distal symmetrical onset, slow progression, absence of fasciculations, and significantly reduced amplitudes on EMG, along with acutely denervated potentials that were localized and symmetrical, we ultimately revised the patient’s diagnosis to dHMN (Ma et al., 2020).

## Discussion

In this report, we presented a case diagnosed dHMN girl harboring a novel mutation within the *SIGMAR1* gene. The genetic affliction manifests an autosomal recessive inheritance pattern, congruent with the observation of a compound heterozygous mutation in our patient.

Although our patient exhibited segmental involvement of both upper and lower motor neurons, which aligns with the diagnostic criteria for ALS, the clinical course presents several atypical features. The patient’s symptoms began in childhood, with relatively rapid progression during the first 4 years, followed by a markedly slow disease progression. The pattern of muscle atrophy displayed a length-dependent distribution, and there was no report of fasciculations. Additionally, the initial EMG showed abnormalities in nerve conduction without the presence of acute denervation potentials. As the disease progressed, the EMG findings evolved to show neurogenic damage characteristic of ALS. However, these denervated potentials were very localized and symmetrically distributed. Notably, although muscle atrophy was not severe, EMG revealed significantly reduced amplitudes, suggesting predominantly axonal involvement. These findings do not support an ALS diagnosis and instead suggest the possibility of dHMN, especially given the prominence of atrophy in both lower limbs. Furthermore, previous reports indicate that ALS associated with *SIGMAR* gene mutations is often accompanied by cognitive impairments, which contrasts with our patient’s normal cognitive function. Research has shown that dHMN primarily affects the peripheral nerves and does not typically impact cognitive function. In summary, we believe the diagnosis of dHMN is more appropriate and aligns better with the family’s psychological expectations (Pareyson and Marchesi, 2009; Kasselimis et al., 2020).

dHMN is a disorder characterized by distal weakness and muscle atrophy. Some phenotypes, as illustrated in this case, also exhibit pyramidal tract signs, suggesting concurrent upper motor neuron dysfunction, which can mimic ALS and lead to misdiagnosis (Ma et al., 2020). A previous report described a consanguineous family with six members carrying a homozygous *SIGMAR1* mutation (Al-Saif et al., 2011). The affected individuals presented with juvenile-onset, symmetrical distal limb weakness, atrophy, and spasticity, and were diagnosed as fALS based on the El Escorial criteria. However, the unusually slow progression of the disease and the absence of fasciculations in these patients are puzzling. Furthermore, it has been suggested that *SIGMAR1* mutations may also cause a classic adult-onset ALS phenotype. However, subsequent findings revealed that one of these patients carried a *C9ORF72* repeat expansion, which further weakens the evidence linking *SIGMAR1* to ALS, suggesting that the association between *SIGMAR1* mutations and familial ALS may be coincidental (Luty et al., 2010; Dobson-Stone et al., 2013; Pickering-Brown and Hardy, 2015). Therefore, we agree with recent studies that classifying *SIGMAR1*-related motor disorders as dHMN, rather than fALS, more accurately reflects the clinical and pathological characteristics of these cases (Ma et al., 2020).

Currently, therapeutic strategies for dHMN associated with *SIGMAR1* gene mutations remain in the investigational phase, with management primarily focused on improving quality of life and slowing disease progression (Frasquet and Sevilla, 2022). As our understanding of these diseases deepens, and recognizing that they differ from ALS in terms of their rapid progression, it is believed that with ongoing advancements in gene therapy, SIGMAR-1 receptor-targeted therapies, and other molecular treatments, more effective therapeutic options may emerge in the future.

## Conclusion

Our case enriches the dHMN database and highlights that adolescent onset, slow progression, length-dependent muscle atrophy, and localized, symmetrical denervation potentials should raise suspicion for dHMN.

## Data Availability

The datasets presented in this article are not readily available because of ethical and privacy restrictions. Requests to access the datasets should be directed to the corresponding author.
