# Supplementary material for: A novel SIGMAR1 missense mutation leads to distal hereditary motor neuropathy phenotype mimicking juvenile ALS: a case report of China
Source: Front Genet. 2025 Apr 16;16:1477518. doi: 10.3389/fgene.2025.1477518 (PMC12041004; doi:10.3389/fgene.2025.1477518)
Supplement: Supplementary file 1 [file Table1.docx]

**Supplementary table S1: EMG data of the patient at the age of nine**

| ***Motor Nerve data*** | | | |
| --- | --- | --- | --- |
| **Nerve and Site** | **Latency (ms)** | **Amplitude (mV)** | **CV (m/s)** |
| Tibial.L (Ankle) | 4.1 | 1.4 | N/A |
| Tibial.L (Popliteal fossa) |  |  |  |
| Peroneal.L (Ankle) |  |  | N/A |
| Peroneal.L (Fibula head) | 13.8 | 0.9 |  |
| Tibial.R (Ankle) |  |  | N/A |
| Tibial.R (Popliteal fossa) |  |  |  |
| Peroneal.R (Ankle) |  |  | N/A |
| Peroneal.R (Fibula head) | 12.5 | 1.4 |  |
| Median.L (Wrist) | 3.7 | 1.8 | 50 |
| Median.L (Elbow) | 7.8 | 1.4 |  |
| Ulnar.L (Wrist) |  |  |  |
| Ulnar.L (Below elbow) |  |  |  |
| Radial.L (Forearm) | 1.8 | 7.6 | 60.4 |
| Radial.L (Forearm) | 4.2 | 9.8 |  |
| Median.R (Wrist) | 3.8 | 1.2 | 45.9 |
| Median.R (Elbow) | 8.7 | 1.2 |  |
| Ulnar.R (Wrist) | 3.3 | 1.6 | 58.9 |
| Ulnar.R (Below elbow) | 7.2 | 1.6 |  |
| Radial.R (Forearm) | 2.2 | 10.3 | 58.3 |
| Radial.R (Forearm) | 4.6 | 5.9 |  |
| **H-waves** | | | |
| Nerve | H-Latency |  |  |
| Tibial.R (Ankle) | N/A |  |  |
| Tibial.L (Ankle) | N/A |  |  |
| ***Sensory Nerve data*** | | | |
| **Nerve and Site** | **Latency (ms)** | **CV (m/s)** |  |
| Sural.L (Ankle) | 2.3 | 43.4 |  |
| Sural.R (Ankle) | 2.4 | 41.6 |  |
| Median.L (Wrist) | 3.2 | 55.3 |  |
| Ulnar.L (Wrist) | 2.7 | 50.0 |  |
| Median.R (Wrist) | 3.2 | 52.2 |  |
| Ulnar.R (Wrist) | 2.7 | 46.2 |  |
| **F-wave** | | |  |
| Nerve | F-Latency |  |  |
| Tibial.L | N/A |  |  |
| Peroneal.L | N/A |  |  |
| Tibial.R | N/A |  |  |
| Peroneal.R | N/A |  |  |
| Median.L | N/A |  |  |
| Ulnar.L | N/A |  |  |
| Radial.L | N/A |  |  |
| Median.R | N/A |  |  |
| Ulnar.R | N/A |  |  |
| Radial.R | N/A |  |  |
| ***MUP data*** | | | |
| Muscle | Mean Amp | Mean Dur | Poly% |
| Left Abd dig min (man) | 128 | 4.1 | 0 |
| Right Abd pollicis brevis | 242 | 6.5 | 0 |
| Left Abd pollicis brevis | 283 | 6.3 | 0 |

MUP: Motor Unit Potential; CV: Conduction Velocity; Amp: Amplitude; Dur: Duration.

| **Supplementary table S2: EMG data of the patient at the age of fourteen** | | | | | | |
| --- | --- | --- | --- | --- | --- | --- |
| ***Motor Nerve data*** | | | | | | |
| Nerve site | Latency (ms) | Amplitude (mV) | Area | Distance (mm) | Interval (ms) | CV (m/s) |
| Median nerve, L |  |  |  |  |  |  |
| Wrist | N/A | N/A | N/A |  | N/A |  |
| Elbow | N/A | N/A | N/A |  | N/A |  |
| Median nerve, R |  |  |  |  |  |  |
| Wrist | N/A | N/A | N/A |  | N/A |  |
| Elbow | N/A | N/A | N/A |  | N/A |  |
| Ulnar nerve, L |  |  |  |  |  |  |
| Wrist | 4.92 | 0.9 | 1.07 |  | 4.92 |  |
| Elbow | 9.18 | 1.25 | 1.44 | 215 | 4.26 | 50.5 |
| Ulnar nerve, R |  |  |  |  |  |  |
| Wrist | 2.94 | 1.3 | 3.6 |  | 2.94 |  |
| Elbow | 6.84 | 0.72 | 2.75 | 225 | 3.9 | 57.7 |
| Tibial nerve, L |  |  |  |  |  |  |
| Around the ankle | N/A | N/A | N/A |  | N/A |  |
| Popliteal fossa | N/A | N/A | N/A |  | N/A |  |
| Tibial nerve, R |  |  |  |  |  |  |
| Around the ankle | N/A | N/A | N/A |  | N/A |  |
| Popliteal fossa | N/A | N/A | N/A |  | N/A |  |
| Fibular nerve, L |  |  |  |  |  |  |
| Around the ankle | N/A | N/A | N/A |  | N/A |  |
| Tibialis anterior | N/A | N/A | N/A |  | N/A |  |
| Fibular head | 3.95 | 1.93 | 11.87 |  | 3.95 |  |
| Fibular nerve, R |  |  |  |  |  |  |
| Around the ankle | N/A | N/A | N/A |  | N/A |  |
| Tibialis anterior | N/A | N/A | N/A |  | N/A |  |
| Fibular head | 3.75 | 2.22 | 15.35 |  | 3.75 |  |
| ***Sensory Nerve data*** | | | | | | |
| Nerve site | Latency (ms) | Amplitude (uV) | Area | Distence (mm) | Interval (ms) | NCV (m/s) |
| Index finger |  |  |  |  |  |  |
| Median nerve, L | 2.4 | 51.5 | 36.96 | 165 | 2.4 | 68.8 |
| Median nerve, R | 2.52 | 38.3 | 31.3 | 160 | 2.52 | 63.5 |
| Little finger |  |  |  |  |  |  |
| Ulnar nerve, L | 2.06 | 46.6 | 29.61 | 130 | 2.52 | 63.1 |
| Ulnar nerve, R | 2.16 | 45.3 | 27.57 | 140 | 2.16 | 64.8 |
| Anterior lateral of the lower leg |  |  |  |  |  |  |
| Superficial fibular nerve, L | 2.34 | 9.7 | 11.78 | 130 | 2.34 | 55.6 |
| Superficial fibular nerve, R | 2.96 | 9.8 | 5.47 | 150 | 2.96 | 50.7 |
| Gastrocnemius nerve |  |  |  |  |  |  |
| Dorsal side of the lower leg, L | 2.5 | 21.2 | 15.79 | 130 | 250 | 52 |
| Dorsal side of the lower leg, R | 3.08 | 11.4 | 7.26 | 150 | 3.08 | 48 |
| ***EMG Summary*** | | | | | | |
| Muscle side | Ins.  Act | Fibrillatory potentials | Pos wave | Dur | Recruit |  |
| Tibialis anterior muscle, R | N/A | ++ | +++ | 15.2 |  |  |
| Tibialis anterior muscle, L | N/A | ++ | +++ | 15.6 |  |  |
| Abductor digiti minimi, R | N/A | N/A | N/A | 16.3 | Mixed phase |  |
| Sternocleidomastoid muscle, R | N/A | N/A | N/A | 11.2 | Interference phase |  |
| Paraspinal muscles, R | N/A | N/A | N/A |  |  |  |

MUP: Motor Unit Potential; CV: Conduction Velocity; Amp: Amplitude; Dur: Duration;
